# Determination of the effective dose of oliceridine combined with propofol using the modified Dixon’s up-and-down method in painless gastroscopy

**DOI:** 10.3389/fphar.2025.1620158

**Published:** 2025-10-27

**Authors:** Jinfeng Cao, Xiaoyu Gu, Xinyang Zhang, Yao Cheng, Liuqin Jiang

**Affiliations:** Department of Anesthesiology, No. 215 Hospital of Shaanxi Nuclear Industry, Xianyang, China

**Keywords:** oliceridine, painless gastroscopy, propofol, effective dose 50, effective dose 95

## Abstract

**Objective:**

To investigate the median effective dose (ED_50_) and 95% effective dose (ED_95_) of oliceridine combined with propofol for painless gastroscopy in adults.

**Methods:**

Patients underwent painless gastroscopy were divided to male and female cohorts. A modified Dixon’s up-and-down sequential method was employed, with an initial oliceridine dose of 20 μg·kg^−1^ for both cohorts. Subsequent dosing adjustments were determined by the procedural success or failure of the preceding patient. The oliceridine dose was increased or decreased by a ratio of 1:1.2 for positive responses or negative responses. We recorded the time of successful induction, examination time, vital signs (HR, SpO_2_ and MAP) at predefined phases (including baseline T_0_, post-induction time T_1_, completion time T_2_, and departure time T_3_), induction dose and total dose of propofol, dose of oliceridine, intraoperative adverse events (including hypoxemia, respiratory depression, hypotension, and bradycardia), postoperative adverse events (including nausea, vomiting, and dizziness), and vasoactive agent administration during the procedure. Probit analysis was subsequently performed to determine the ED_50_, ED_95_ and corresponding 95% confidence intervals (CIs) of oliceridine in painless gastroscopy combined with propofol.

**Results:**

The ED_50_ and ED_95_ of oliceridine combined with propofol were determined as 12.63 μg·kg^−1^ (95% CI: 11.43–13.79) and 14.46 μg·kg^−1^ (95% CI: 13.41–20.33) in males, and 10.38 μg·kg^−1^ (95% CI: 9.02–11.96) and 13.19 μg·kg^−1^ (95% CI: 11.62–28.23) in females. Male negative subgroup required higher oliceridine doses (P < 0.05), while female negative subgroup had lower total propofol dose yet higher oliceridine doses (P < 0.05). Females in the negative subgroup used more propofol (P < 0.05), and both sexes’ negative subgroups consumed more oliceridine (P < 0.05). In males, SpO_2_ rose at T_1_ and T_2_ (P < 0.01), and MAP dropped at T_2_ and T_3_ (P < 0.05). In females, HR decreased at T_2_ (P < 0.05), SpO_2_ increased at T_1_ (P < 0.05), and MAP fell at T_2_ and T_3_ (P < 0.05). Adverse events included postoperative dizziness (12.50%), nausea (4.17%), and fatigue (4.17%) in females, and vomiting (5.56%) in males.

**Conclusion:**

The use of oliceridine (13.19–14.46 μg·kg^−1^) and propofol was associated with safety, efficacy, and lower complication rates during painless gastroscopy.

**Clinical Trial Registration:**

https://www.chictr.org.cn/showproj.html?proj=249883, identifier ChiCTR2400093609

## 1 Introduction

Gastroscopy is the gold standard for diagnosing upper gastrointestinal diseases and can effectively detect conditions such as gastritis, gastric polyps, and gastric or esophageal tumors. Relevant epidemiological studies indicate that gastroscopy is recommended for the general population aged ≥45 years, and individuals with normal findings are advised to undergo repeat gastroscopy every 3–5 years, while patients diagnosed with chronic atrophic gastritis or higher-grade lesions should receive annual endoscopic surveillance (Li et al., 2024). Nevertheless, the clinical utility of conventional gastroscopy is compromised by procedure-induced viscerosensory reactions (including emesis, laryngospasm, and autonomic instability), which contribute to suboptimal adherence. Among patients undergoing gastroscopy, 41%–61% exhibited clinically significant procedural discomfort and anxiety (Abraham et al., 2002). With advancements in medical technology and heightened health awareness, painless gastroscopy has gained increasing clinical adoption. Sedated gastroscopy maintains patients in a sleep state throughout the procedure, effectively eliminating anxiety and pain during procedure. This approach not only enhances endoscopic visualization quality but also facilitates the detection of subtle early-stage lesions and improves diagnostic accuracy. The current pharmacological mainstay predominantly utilizes propofol combined with opioids. Propofol demonstrates favorable sedative properties, including rapid onset, short duration of action, and predictable recovery profiles. However, its limited analgesic efficacy and inability to suppress stress responses necessitate dose escalation, which may induce dose-dependent hypotension and respiratory complications (e.g., shallow breathing, bradypnea, or transient apnea) (Martorano et al., 2008). Consequently, adjunct administration with opioid analgesics remains standard practice. Nevertheless, the concomitant use of traditional opioids such as fentanyl elevates risks of opioid-related adverse events, particularly nausea/vomiting and respiratory depression (Tsai et al., 2021). It was demonstrated that the incidence of opioid-related adverse events (ORAE) has been reported to reach 10.6%, which is associated with prolonged hospital stays, increased in-hospital mortality, elevated healthcare expenditures, and higher 30-day readmission rates (Shafi et al., 2018).

Oliceridine is a novel opioid analgesic that functions as a G protein-biased μ-opioid receptor agonist. It provides effective analgesia while demonstrating a reduced incidence of adverse effects such as respiratory depression and gastrointestinal dysfunction compared to conventional opioids (Simons et al., 2023). Based on oliceridines’ pharmacokinetic profile, intravenous bolus administration achieves rapid onset of action within 1–2 min, peaks at 6–12 min, and maintains therapeutic effects for approximately 1–3 h (Kaye et al., 2021). The drug is primarily metabolized by cytochrome P450 (CYP) hepatic enzymes, with inactive metabolites exhibiting negligible pharmacological activity. Pharmacokinetic studies reveal comparable clearance between patients with hepatic or renal impairment and those with normal organ function, supporting its safety in these populations (Nafziger et al., 2020). However, dose reduction may be required for moderate-to-severe hepatic impairment during prolonged administration (Gan and Wase, 2020). These characteristics make oliceridine not only suitable for acute pain management but also advantageous for short-duration procedures such as gynecological interventions and gastrointestinal endoscopy.

Oliceridine demonstrates potent analgesic efficacy with a favorable adverse effect profile characterized by reduced incidence of respiratory depression and gastrointestinal dysfunction. This pharmacological advantage suggests that its combination with propofol may establish a safer and more effective paradigm for painless diagnostic or therapeutic procedures. However, current evidence on oliceridine utilization in non-operating room anesthesia (NORA) contexts remains limited. The safety and efficacy profiles have not been fully characterized owing to a paucity of data regarding clinically validated dosing regimens, and no studies to date have systematically investigated potential sex-specific variations in therapeutic outcomes. This study therefore quantified the median effective dose (ED_50_) and 95% effective dose (ED_95_) of oliceridine-propofol coadministration stratified by biological sex during procedural sedation. The established dose-response profiles provide pharmacodynamic references to optimize gender-tailored oliceridine administration in clinical practice.

## 2 Methods

### 2.1 Ethics and trial registration

Written informed consent was obtained from all enrolled participants, with explicit assurance of voluntary withdrawal rights throughout the trial duration. The study protocol received ethical endorsement from the Institutional Review Board (IRB) of the ethics committee at NO.215 Hospital of Shaanxi Nuclear Industry [Approval No. 2024(030)] and was prospectively registered in the ClinicalTrials.gov registry (Registration ID: ChiCTR2400093609).

### 2.2 Patients

Patients scheduled for painless gastroscopy between January to March 2025 were screened. Inclusion criteria: (1) age 18–64 years; (2) BMI 18.5–29.9 kg/m^2^; (3) ASA physical status I to II; (4) indicated for diagnostic painless gastroscopy. Exclusion criteria: (1) severe cardiopulmonary, cerebrovascular, hepatic, or renal comorbidities; (2) history of documented hypersensitivity to anesthetic agents or excipients; (3) requirement for advanced endoscopic interventions. Elimination criteria: (1) occurrence of severe anesthesia- or procedure-related complications; (2) voluntary withdrawal from the study.

### 2.3 Study interventions

All participants adhered to standardized preoperative fasting protocols (8 h for solids, 3 h for clear liquids) without premedication. Upon entering the procedure suite, intravenous access was established with continuous monitoring of non-invasive blood pressure (NIBP), electrocardiogram (ECG), and peripheral oxygen saturation (SpO_2_). Preoxygenation was administered via an endoscopy-specific facemask at 5 L/min. Sedation was initiated with intravenous (IV) oliceridine (Jiangsu Nhwa Pharmaceutical Co., Ltd., Jiangsu, China) over 2 min, followed by propofol (Guangdong Jiabo Pharmaceutical Co., Ltd., Guangdong, China) delivered at 40 mg/10 s. Endoscopic insertion commenced when the Observer’s Assessment of Alertness/Sedation (OAA/S) scale (score 5: responds readily to verbal commands in normal tone; score 4: lethargic but appropriate response to commands in normal tone; score 3: responds only after name is called repeatedly and/or loudly and requires tactile stimulation to elicit movement; score 2: responds only to mild noxious prodding or shaking; score 1: no response and motor reflexes to painful stimuli) reached ≤1. Preliminary trials indicated that a propofol dosage range of 1–1.5 mg·kg^−1^ was required to achieve OAA/S score ≤1 in patients. Hemodynamic support: dopamine (1–2 mg IV) was given intravenously for mean arterial pressure (MAP) < 60 mmHg or systolic blood pressure decreased 20% of baseline; Atropine (0.5 mg IV) for heart rate (HR) < 50 bpm; Jaw-thrust maneuver with supplemental oxygen for oxygen saturation (SpO_2_) <90%. All procedures were performed by a single attending anesthesiologist and the same gastroenterologist.

### 2.4 Modified Dixon’s up-and-down sequential method

Enrolled patients were stratified into male and female cohorts. Based on pre-trial pharmacokinetic and morphine milligram equivalent (MME) conversion analysis, the initial oliceridine dose was set at 20 μg·kg^−1^ (equivalent for both sexes), with this regimen demonstrating equipotent analgesic efficacy to 0.1 μg·kg^−1^ sufentanil. The oliceridine dose was increased or decreased by a ratio of 1:1.2 (Xu et al., 2023) for positive responses (failure) or negative responses (success). Subsequent dosing adjustments were determined by the procedural success/failure of the preceding patient. The formal test commenced at the first crossover point and terminated upon observing seven consecutive reversals (Pace and Stylianou, 2007). Positive response was defined as (Feng et al., 2019): during endoscope insertion or within 3 min of pharyngeal entry, (1) body movement or cough ≥ grade 2 (purposeful limb movement or persistent cough requiring temporary suspension of the procedure); or (2) blood pressure or heart rate exceeding 30% of baseline values. Positive responders requiring sedation initially received propofol (0.5 mg·kg^−1^ IV bolus; maximum two cumulative doses). If inadequate gastroscopic conditions persisted, a rescue regimen of sufentanil (Yichang Renfu Pharmaceutical Co., Ltd., Hubei, China, 0.05 μg·kg^−1^ IV) co-administered with propofol (1 mg·kg^−1^ IV) was administered as an adjunctive analgesic protocol. All patients were transferred to the post-anesthesia care unit (PACU) for recovery, where anesthesia nurses continuously monitored vital signs. Patients were discharged from the PACU upon achieving a modified Aldrete score of ≥9. All cases underwent follow-up within 24 h.

### 2.5 Statistical analysis

Statistical analyses were performed using SPSS Statistics version 27.0 (IBM Corp.). Normally distributed continuous variables are presented as mean ± standard deviation (SD), with between-group comparisons conducted via independent Student's t-tests. Non-normally distributed data are expressed as median (interquartile range, IQR) and analyzed using Mann-Whitney U tests. The categorical data were subjected to analysis using the chi-square test. Post hoc pairwise comparisons among multiple groups were performed using one-way analysis of variance (ANOVA). Probit regression analysis was employed to calculate the ED50 and ED95 of oliceridine for painless gastroscopy, with corresponding 95% confidence intervals (95% CI). Dose-response curves and sequential trial plots were generated using GraphPad Prism version 5.0 (GraphPad Software Inc.). A two-tailed p-value <0.05 defined statistical significance.

## 3 Outcomes

### 3.1 Primary outcomes

The ED_50_ and ED_95_ of oliceridine with 95% CI combined with propofol for painless gastroscopy in male and female cohort.

### 3.2 Other outcomes

Demographic and clinical baseline characteristics included age, height, weight, BMI, ASA physical status and basic vital signs.

Induction dose and total administered dose of propofol, dose of oliceridine, time to successful induction, gastroscopy duration (from scope insertion to withdrawal) and administration of vasoactive agents were recorded.

HR, SpO_2_ and MAP were monitored at predefined intervals: baseline (T_0_), post-induction time (T_1_), completion time (T_2_) and departure time (T_3_).

Adverse events were categorized as:

1. Intraoperative: hypoxemia (SpO_2_ <90% for >30s), respiratory depression (respiratory rate <8 breaths/min), hypotension (MAP <60 mmHg or systolic blood pressure decreased >20% of baseline), bradycardia (HR < 50 bpm).
2. Postoperative: nausea, vomiting, dizziness and fatigue after discharge.

## 4 Results

**FIGURE 1:**
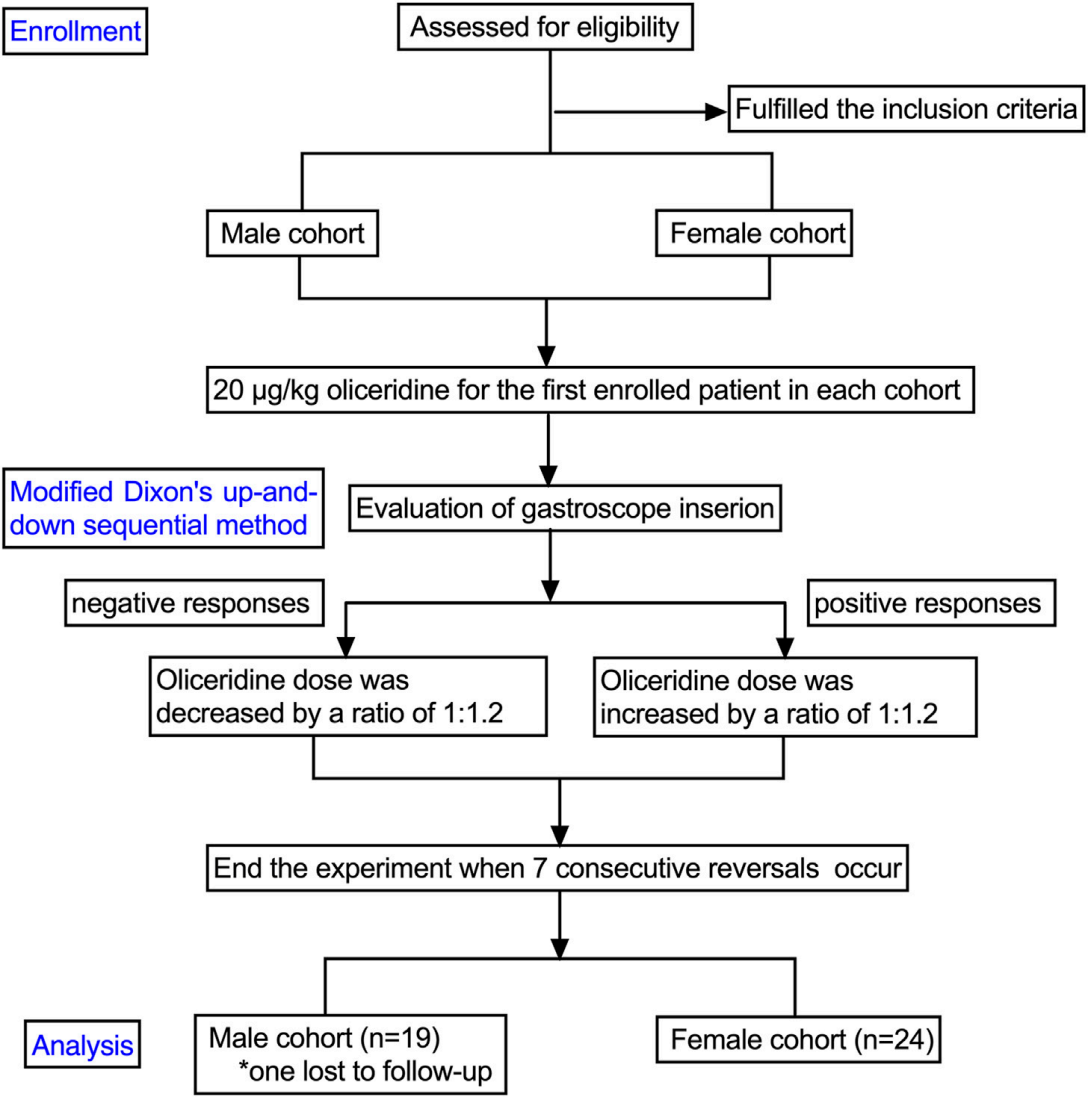
The flowchart of the study. Participants are assessed for eligibility, with those fulling criteria divided into male and female cohorts. Both receive 20 micrograms per kilogram of oliceridine. Using a modified Dixon’s method, responses to gastroscope insertion are evaluated. Negative responses prompt a 1:1.2 dose reduction; positive responses lead to a 1:1.2 increase. The experiment concludes after seven consecutive reversals. Final analysis includes 19 males, with one lost to follow-up, and 24 females.

The study cohort comprised 24 female patients and 19 male patients. One participant in the male group was lost to follow-up after endoscopic procedure (Figure 1). The baseline characteristics of the patients are summarized in Table 1. No statistically significant differences in baseline characteristics were observed between positive and negative subgroups within either sex-stratified cohort (P > 0.05). In the male cohort, patients in the negative group required higher doses of oliceridine compared to the positive group (P < 0.05). In the female cohort, the total propofol dose was lower in the negative group than in the positive group (P < 0.05), while oliceridine doses was higher in the negative group (P < 0.05) (Table 1).

**FIGURE 2:**
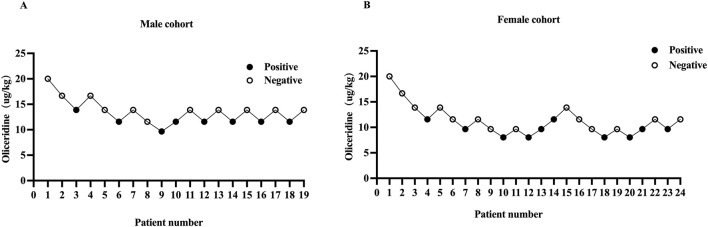
Individual responce to the stimulus of gastroscopy placement in male **(A)** and female **(B)** cohorts. The black dots represent “positive” responses and the white dots represent “negative” responses.

**FIGURE 3:**
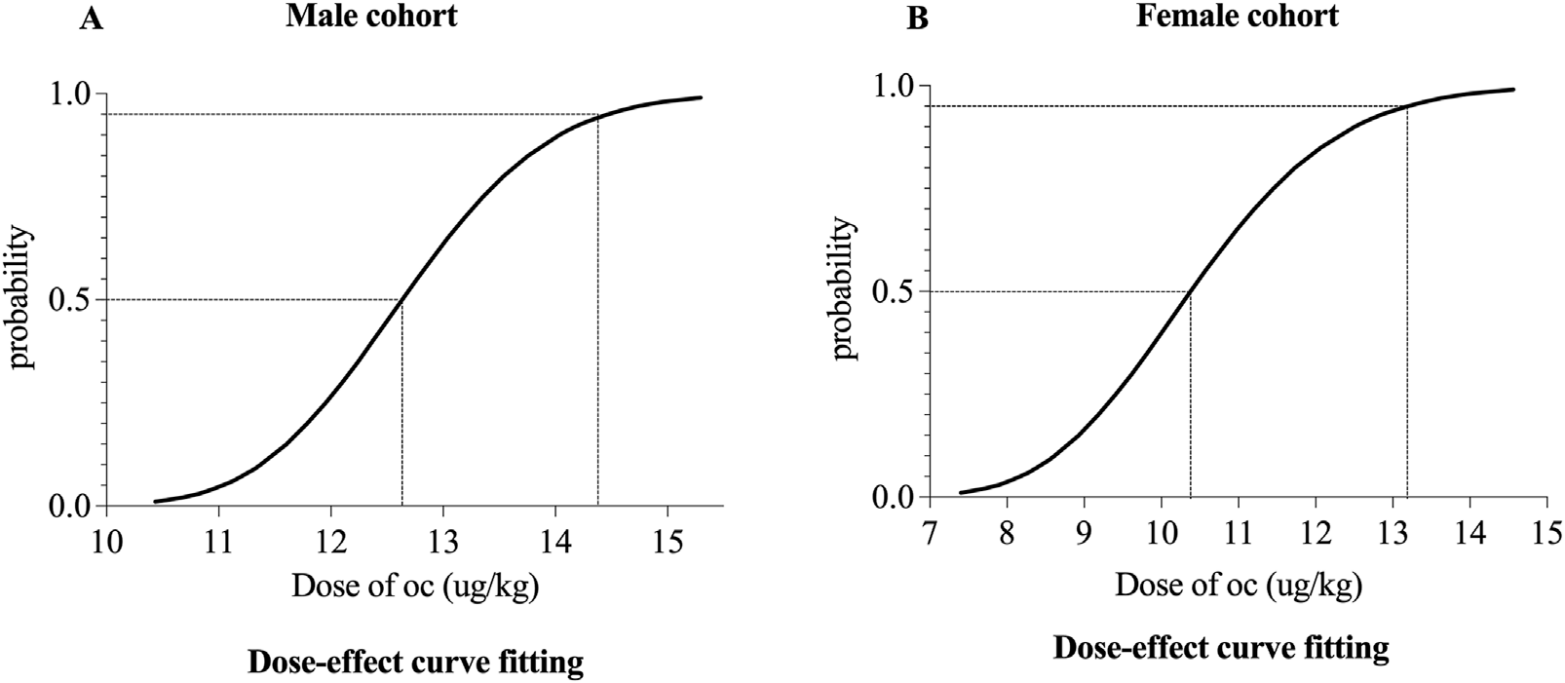
Dose–response curve of oliceridine in painless gastroscopy for male **(A)** and female **(B)** cohorts.

1. Our study was conducted until data for seven crossover points were collected (Figure 2). The ED_50_ and ED_95_ values of oliceridine fumarate combined with propofol in painless gastroscopy were 10.38 μg·kg^−1^ (95% CI: 9.02–11.96) and 13.19 μg·kg^-1^ (95% CI: 11.62–28.23) in the female group, and 12.63 μg·kg^−1^ (95% CI: 11.43–13.79) and 14.46 μg·kg^−1^ (95% CI: 13.41–20.33) in the male group, respectively (Figure 3).
2. No significant differences were observed in the time to successful induction, gastroscopy duration, or propofol induction dose between positive and negative subgroups within male and female cohorts (P > 0.05). In the female cohort, the total propofol dose was significantly higher in the negative subgroup compared to the positive subgroup (P < 0.05). No statistically significant difference in total propofol consumption was observed between negative and positive subgroups within the male cohort(P > 0.05). Oliceridine consumption was significantly higher in negative subgroups compared to positive subgroups within both male and female cohorts (P < 0.05) (Table 1).
3. In male cohorts, compared to T_0_, SpO_2_ significantly increased at T_1_ and T_2_ (P < 0.01), while MAP decreased at T_2_ and T_3_ (P < 0.05). In female cohorts, compared to T_0_, HR significantly decreased at T_2_ (P < 0.05), SpO_2_ increased at T_1_ (P < 0.05), and MAP decreased at T_2_ and T_3_ (P < 0.05). None of the patients required vasoactive drugs during the study period (Table 2).
4. Adverse events

**TABLE 1:** Characteristics of patients.

Parameter	Male cohort (n = 19)		*t/Z/χ* ^*2*^ value	*P* value	Female cohort (n = 24)		*t/Z/χ* ^*2*^ value	*P* value
	Positive	Negative			Positive	Negative		
Age (years)	46.75 (10.22)	49.45 (13.03)	*t* (0.49)	0.63	49.80 (11.21)	45.36 (9.01)	*t* (1.08)	0.29
Height (cm)	172.13 (3.72)	172.36 (4.13)	*t* (0.13)	0.90	160.40 (4.72)	158.50 (3.52)	*t* (1.13)	0.27
Weight (kg)	72.00 (8.21)	72.45 (5.32)	*t* (0.15)	0.89	57.70 (4.81)	56.00 (6.97)	*t* (0.66)	0.51
BMI (kg/m^2^)	24.24 (2.38)	24.53 (1.89)	*t* (0.30)	0.77	22.40 (1.11)	22.43 (2.53)	*t* (0.04)	0.97
ASA								
I (%)	0	2	*χ 2* (1.63)	0.20	2	4	χ^2^ (0.23)	0.63
II (%)	8	9			8	10		
Induction time (min), Median (IQR)	3.00 (0.75)	3.00 (0)	*Z* (0.82)	0.42	3.00 (1.25)	3.00 (1.00)	*Z* (1.00)	0.32
Gastroscopy duration (min)	5.38 (2.20)	7.45 (3.08)	*t* (1.63)	0.12	5.50 (2.25)	5.00 (1.00)	*Z* (0.62)	0.54
Induction propofol dosage (mg), Median (IQR)	88.75 (13.56)	90.00 (7,75)	*t* (0.26)	0.80	74.00 (14.30)	73.93 (8.13)	*t* (0.02)	0.99
Total propofol dosage (mg), Median (IQR)	133.13 (16.68)	146.36 (33.25)	*t* (1.03)	0.32	128.00 (39.38)	99.29 (19.70)	*t* (2.36)	0.03*
Oliceridine dosage (mg), Median (IQR)	11.57 (0.00)	13.89 (2.78)	*Z* (3.22)	0.00*	9.65 (2.09)	11.57 (4.24)	*Z* (2.97)	0.00*

Data are presented as the mean ± SD, median (interquartile range) or as the number (proportion) as appropriate. Two-sided *t*-test, *U*-text, or *Chi-square* test, *P < 0.05.

**TABLE 2:** HR, SpO_2_ and MAP at different time points for patients.

Parameter	Male cohort (n = 19)	*P*	Female cohort (n = 24)	*P*
HR (bmp)				
T_0_	77.63 (10.53)		80.50 (8.72)	
T_1_	75.00 (8.76)	1.00	73.15 (9.13)	0.08
T_2_	75.89 (8.41)	1.00	72.25 (6.22)	0.03*
T_3_	76.63 (7.89)	1.00	76.17 (8.78)	1.00
SpO2 (%)				
T_0_	97.21 (1.58)		97.71 (1.38)	
T_1_	99.16 (1.54)	0.00*	99.18 (1.29)	0.02*
T_2_	98.84 (1.54)	0.02*	98.96 (1.30)	0.11
T_3_	97.42 (1.71)	1.00	97.63 (2.04)	1.00
MAP (mmHg)				
T_0_	92.68 (11.04)		90.79 (10.81)	
T_1_	84.21 (13.07)	0.21	87.00 (12.93)	1.00
T_2_	77.00 (9.86)	0.00*	74.46 (11.02)	0.00*
T_3_	78.42 (10.22)	0.00*	76.96 (10.21)	0.00*

Data are presented as mean ± SD or number. Significance for *post hoc* analysis after analysis of variance (ANOVA) was corrected with Bonferroni’s method. Compared with T_0_ *P < 0.05. T_0_ is the time before the administration of oliceridine (baseline); T_1_ is the time of post-induction; T_2_ is the time of completion; T_3_ is the time of departure.

No significant intraoperative complications, including hypotension, bradycardia, or respiratory depression, were observed in any patient during the procedure. Male patients exhibited vomiting (1/18, 5.56%) and one male participant was lost to follow-up during the study period. Among female, postoperative adverse events included dizziness (3/24, 12.50%), nausea (1/24, 4.17%), and fatigue (1/24, 4.17%) (Table 3).

**TABLE 3:** Postoperative adverse events data.

Adverse event	Male cohort (n = 18)	Female cohort (n = 24)	*P*
Dizziness	0	3 (12.50%)	0.25
Nausea	0	1 (4.17%)	1.00
Vomiting	1 (5.56%)	0	0.43
Fatigue	0	1 (4.17%)	1.00

Chi-squared test and Fisher’s exact test were used to evaluate the differences between two cohorts.

## 5 Discussion

Our study established the effective dose of oliceridine combined with propofol for gastroscopic procedures. Probit regression analysis determined the ED_50_ and ED_95_ of oliceridine to suppress endoscope insertion responses as 10.38 and 13.19 μg·kg^−1^ in females, 12.63 and 14.46 μg·kg^−1^ in males. All patients safely and effectively completed the examination without serious complications. Jia et al. (2024) demonstrated that the ED_50_ of oliceridine combined with propofol for suppressing gastroscope insertion responses was 15 μg·kg^−1^ in patients aged 18–65 years, compared with 12 μg·kg^−1^ in those aged >65 years. These findings are similar with our observations. The study by Tang et al. (Tang et al., 2025) reported ED_90_ and ED_99_ values of 22.5 and 23.8 μg·kg^−1^ for oliceridine. The observed discrepancies in our findings may be attributed to methodological variations, particularly their use of the biased coin design (BCD), which potentially enhances the precision of ED_90_ determination compared to our approach.

Previous studies have demonstrated sex-related differences in the analgesic efficacy of various opioid analgesics. Our findings revealed that both ED_50_ and ED_95_ values were significantly lower in the female cohort compared to the male cohort. The mechanisms underlying these sex-based disparities in opioid efficacy are multifactorial. Key contributors include differential pain sensitivity, sex-specific nociceptive signal processing, and variations in drug concentration requirements, receptor sensitivity, and gonadal hormone modulation (Bradbury, 2003; Chaudhary et al., 2017). Notably, neuroimaging evidence indicates that females exhibit stronger μ-opioid receptor binding capacity in cortical and subcortical regions, enabling enhanced analgesic responses to equivalent opioid doses (Bodnar and Kest, 2010). Furthermore, heightened opioid sensitivity in females may stem from gonadal hormone-mediated regulation of opioid receptors (Joe et al., 2016). These hormonal interactions potentiate the inhibitory effects of opioids on stress responses in female patients, underscoring the necessity for sex-specific considerations in clinical analgesic regimens. In clinical practice, individualized dose adjustments of oliceridine should be implemented based on patients’ sex, body weight, pain intensity, and previous medication responses, with particular attention to the potential requirement for lower initial doses in female populations. Close monitoring of therapeutic responses, especially in female patients, remains imperative. These tailored therapeutic strategies and vigilant surveillance constitute the cornerstone for ensuring optimal drug safety and efficacy.

Oliceridine dosage requirements exhibit significant variation across different clinical procedures. Zhang et al. (2024) reported that the ED_50_ of oliceridine combined with 2 mg·kg^−1^ propofol for analgesia during induced abortion was 19 μg·kg^−1^ (95% CI: 15–23 μg·kg^−1^) in patients with a history of vaginal delivery, compared to 26 μg·kg^−1^ (95% CI: 21–31 μg·kg^−1^) in those without such history. Compared to gastroscopy, induced abortion procedures induce more intense nociceptive stimulation, thus necessitating higher oliceridine doses to achieve adequate pain control.

Oliceridine demonstrates approximately 5-fold greater analgesic potency than morphine, effectively suppressing gag reflex, cough, and cardiovascular adverse responses triggered by gastroscope passage through the oropharynx. When combined with propofol, it reduces propofol requirements while mitigating the risk of severe respiratory depression. No procedure-related adverse events (respiratory depression, hypotension, or bradycardia) were observed in the study cohort. This safety profile may be attributed to two key factors: (1) oliceridine’s minimal respiratory depressive effects, as evidenced by prior studies showing comparable respiratory depression rates between oliceridine and placebo even in high-risk populations (BMI ≥30 kg/m^2^) (Brzezinski et al., 2021); (2) All patients in this study were administered oxygen via dedicated endoscopic masks, which potentially contributed to the reduced incidence of hypoxemia.

Oliceridine has gained significant interest for demonstrating a reduced risk of adverse events (e.g., respiratory depression and gastrointestinal complications) relative to conventional opioids, thereby positioning itself as a potential therapeutic breakthrough to mitigate the clinical challenges associated with existing opioid regimens (Wang et al., 2024). A systematic review and meta-analysis of randomized controlled trials has shown that oliceridine exhibits robust therapeutic efficacy and a favorable safety profile as an intravenous analgesic for postoperative pain management, delivering prompt pain relief with superior tolerability and a significantly lower risk of adverse events relative to morphine and hydromorphone (Biskupiak et al., 2024; Niu et al., 2023). In our study, nausea and vomiting were reported in both male and female cohorts, with a higher incidence observed among female participants. This disparity may be associated with sex-specific physiological characteristics in females, potentially linked to differential pharmacokinetic responses or estrogen-mediated visceral sensitivity modulation.

Postoperative adverse events were infrequent: dizziness (3/24, 12.5%), nausea (1/24, 4.2%), and fatigue (1/24, 4.2%) in females; vomiting (1/19, 5.3%) and one loss to follow-up in males. These findings align with oliceridine’s biased μ-opioid receptor agonism-preferentially activating G protein-coupled signaling pathways while minimizing β-arrestin recruitment-thereby reducing classic opioid-related adverse effects without compromising analgesia (Bergese et al., 2019; Sun et al., 2023).

This study has the following limitations. Firstly, the research was constrained by the modified sequential method and its single-center design, leading to a relatively limited final sample size. Adopting multicenter studies with larger sample sizes would enhance the accuracy of the findings. Secondly, patients with comorbid chronic conditions were not included in this investigation. The anesthetic efficacy and safety of propofol combined with oliceridine in populations with underlying chronic diseases require further exploration. Thirdly, this study did not employ end-tidal carbon dioxide (PetCO_2_) monitoring. Future research incorporating PetCO_2_ monitoring could more sensitively and precisely reveal the dynamic changes in respiratory depression during sedation and its independent association with adverse reactions such as hemodynamic changes. Fourthly, additional randomized controlled trials are warranted to investigate the clinical applications of oliceridine in anesthesia practice.

## 6 Conclusion

The combination of oliceridine and propofol effectively suppresses the response to gastroscope insertion during painless gastroscopy, with high safety and minimal complications. In the female group, the ED_50_ of oliceridine was 10.38 μg·kg^−1^, and the ED_95_ was 13.19 μg·kg^−1^. In the male group, the ED_50_ of oliceridine was 12.63 μg·kg^−1^, and the ED_9_5 was 14.46 μg·kg^−1^.

## Data Availability

The original contributions presented in the study are included in the article/Supplementary Material, further inquiries can be directed to the corresponding author.
